# Description of evandromyia spelunca, a new phlebotomine species of the *cortelezzii *complex, from a cave in Minas Gerais State, Brazil (Diptera: Psychodidae: Phlebotominae)

**DOI:** 10.1186/1756-3305-4-158

**Published:** 2011-08-09

**Authors:** Gustavo ML Carvalho, Reginaldo P Brazil, Cristiani C Sanguinette, José D Andrade Filho

**Affiliations:** 1 Coleção de Flebotomíneos, Centro de Referência Nacional e Internacional para Flebotomíneos, Instituto René Rachou, FIOCRUZ, Av. Augusto de Lima 1715, 30190-002 Belo Horizonte, MG, Brazil; 2 Departamento de Bioquímica e Biologia Molecular, Instituto Oswaldo Cruz, FIOCRUZ, Rio de Janeiro, RJ, Brazil

## Abstract

**Background:**

The cave fauna of the Brazil is poorly documented, and among the insects those live or frequent caves and their adjacent environments phlebotomine sand flies call for special attention because several species are vectors of pathogens among vertebrates hosts. A new species of sand fly from Minas Gerais is described based in females and males collected in a cave of the municipality of Lassance.

**Results:**

The morphological characters of the new species permit to include in the Evandromyia genus, cortelezzii complex. This complex consists of three species: Evandromyia corumbaensis (Galati, Nunes, Oshiro & Rego, 1989), Evandromyia cortelezzii (Brethes, 1923) and Evandromyia sallesi (Galvao & Coutinho, 1940).

**Conclusions:**

The new species can be separate from the others of the cortelezzii complex through morphological characters of the male terminalia and female spermathecae.

## Background

Sand flies are responsible for transmission of the genus *Leishmania *among vertebrates hosts and study of this insect group is of great importance in attempts to control of leishmaniasis [1]. In Brazil, the cave fauna of insects is poorly documented, and among the insects that live or frequent caves and their adjacent environments, phlebotomine sand flies call for special attention because several species are vectors of arboviruses and protozoa, among other parasites.

The geographical distribution of the sand flies species depends on their ability to adapt to different ecological niches. Thus, by its development in the immature stages and their feeding habits when adults, the species of sand flies are found where both larvae and adults can find appropriate environments for their development, including blood supply for females. In this way, the geographical distribution of species can be restricted to their access to a specific environment and vertebrate host. Already the occurrence of leishmaniasis is basically determined by the presence of both a susceptible vector and a host/reservoir equally susceptible to the infection [2].

The growing of tourism, involving a search for natural attractions such as cave exploration, demands a better knowledge of the threats to health that people may face in those new areas.

In the present investigation a new species of sand fly from Minas Gerais is described based on both genders collected in a cave in the Lassance municipality.

## Methods

Sand flies were collected using CDC light traps (HP model), conducted between June 2008 to May 2010, in the cave named "Gruta Rebenta Bombas" city of Lassance, Minas Gerais state. Sand flies were mounted in Canada balsam (males) and Berlese liquid (females), measured with a binocular Olympus CH-2 microscope with the aid of a micrometer ocular and the drawings were done with the help of a *camera lucida*. All the measurements presented in this paper are given in micrometers. The nomenclature and classification is that proposed by Galati [3] and the abbreviation of the names for phlebotomine genera by Marcondes [4]. The new species is described based on 10 females and 10 males, which were associated by their capture in the same place at the same time as well as by the association of the morphological characteristics (antennal and palpal formulas; length association of the genital filaments; pigmentation of the thorax; and other structures) that include all the specimens of both sexes in the same genus and group.

In accordance with section 8.6 of the ICZN's International Code of Zoological Nomenclature, copies of this article are deposited at the following five publicly accessible libraries: Natural History Museum, London, UK; American Museum of Natural History, New York, USA; Museum National d'Histoire Naturelle, Paris, France; Russian Academy of Sciences, Moscow, Russia; Academia Sinica, Taipei, Taiwan.

### Description of *Evandromyia spelunca *-nov.sp. (Figures 1-12)

#### Holotype male

sand fly of medium size, measurement *ca*. 2,677 (2,609 ± 125.4; n = 10) in length, general colour light brown.

**Figure 1:**
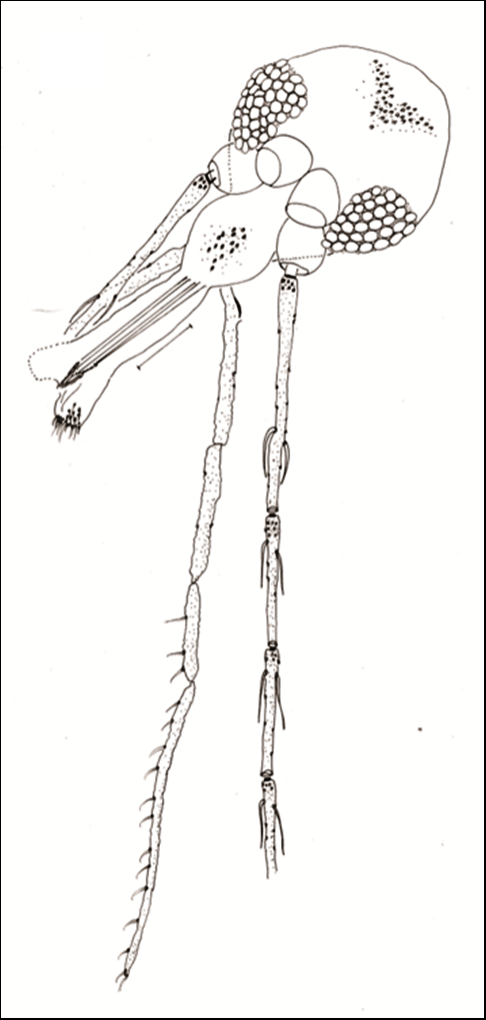
***Evandromyia spelunca *sp. nov. (Holotype male)**. Head, frontal view. Bar = 100 μm.

#### Head (Figure 1)

386 (370 ± 13.9; n = 10) long and 262 (261 ± 10.0; n = 10) wide. Head length/head width ratio 1.47: 1 (1.42 ± 0.08; n = 10). Clypeus 133 (129 ± 7.1; n = 10) long; clypeus length/head length ratio 0.34: 1 (0.35: 1 ± 0.02; n = 10). Eye 126 (132 ± 16.9; n = 10) long and 75 (83 ± 10.7; n = 10) wide; eye length/head length 0.33: 1 (0.36: 1 ± 0.04; n = 10). Interocular distance 119 (114 ± 6.8; n = 10). Labrum-epipharynx (LE) 218 (215 ± 5.8; n = 10). LE/head length 0.56: 1 (0.58 ± 0.01; n = 10). Labial suture forming a fork. Antenna with simple and short ascoid, not reaching the next flagellomere. Antennal formula AIII-AXV: 2 Antennomere lengths: AIII 304 (297 ± 10.0; n = 10); AIV 166 (155 ± 8.4; n = 10); AV 166 (157 ± 7.5; n = 10); AXV > AXVI (AXV > AXVI; n = 7). AIII, AIV, AV, AXII, AXIII, AXIV, AXV, AXVI with papilla; ratios: AIII/head length 0.79: 1 (0.80: 1 ± 0.04; n = 10); AIII/LE 1.39: 1 (1.38: 1 ± 0.06; n = 10). Palpal formula 1.4.2.3.5 (1.4.2.3.5; n = 10). Palpomere lengths: P1 37 (36 ± 3.3; n = 10); P2 146 (139 ± 4.1; n = 10); P3 173 (167 ± 6.0; n = 10); P4 122 (113 ± 5.7; n = 10); P5 400 (367 ± 43.6; n = 10). Newstead's spines inserted medially on palpomere 3, also present on its apical third portion.

#### Cervix

Ventrocervical sensillae: present

#### Thorax

**Figure 2:**
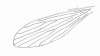
***Evandromyia spelunca *sp. nov. (holotype male)**. Wing. Bar = 250 μm.

Proepimeral setae present, 4-4 [(4-4; n = 2), (3-4; n = 3), (4-5; n = 2), (3-5; n = 1), (3-3; n = 1)] and anepisternal superior setae present, 9-9 [(12-12; n = 2), (13-13; n = 2), (9-10; n = 2), (13-14; n = 1), (10-12; n = 1), (10-10; n = 1)]; setae on the anterior region of the katepisternum present. Wing (Figure 2) measurement 1,973 (1,907 ± 50.7; n = 10) long and 483 (471 ± 59.7; n = 10) at maximum width. Length/width ratio 4.08: 1 (4.05: 1 ± 0.37; n = 10). Length of the vein sections: R_5 _1,242 (1,213 ± 29.5; n = 10); *alpha *359 (336 ± 37.0; n = 10); *beta *276 (258 ± 14.7; n = 10); *gamma *649 (625 ± 25.4; n = 10); *delta *41 (59 ± 25.3; n = 10). Legs: anterior, median and posterior, respectively: femur 731 (727 ± 15.2; n = 9), 662 (690 ± 23.6; n = 10) and 787 (764 ± 25.7; n = 9); tibia 952 (924 ± 35.3; n = 9), 1,090 (1,047 ± 68.0; n = 10) and 1,256 (1,267 ± 17.9; n = 9); tarsomere I 580 (560 ± 22.1; n = 9), 635 (621 ± 18.7; n = 10) and 690 (698 ± 24.1; n = 9); tarsomeres II+III+IV+V 676 (635 ± 33.1; n = 9), 690 (661 ± 21.9; n = 10) and 745 (718 ± 24.4; n = 8).

#### Abdomen

**Figure 3:**
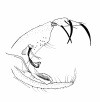
***Evandromyia spelunca *sp. nov. (holotype male)**. Terminalia. Bar = 100 μm.

**Figure 4:**
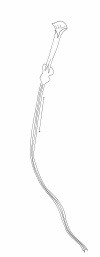
***Evandromyia spelunca *sp. nov. (holotype male)**. Genital pump and filaments. Bar = 100 μm.

terminalia (Figure 3): gonostyle 133 (133 ± 3.5; n = 10) long, with four spines: one apical, one upper external, inserted in a tubercle near the outer lower, which is thinner than that, and an internal implanted below the outside and with the same caliber of this. Sub terminal seta present. Gonocoxite 245 (231 ± 13.7; n = 10) long and 92 (89 ± 7.7; n = 10) wide, with a tuft containing four long and slender setae, inserted in a small tubercle, and two smaller setae, inserted beside this others. The base of the tuft presents a small pigmented area. Paramere thickened at the base, which narrows in the middle region, and widens again, taking the shape of a bird's head with the nozzle facing down. This paramere presents a suture that divides the inside and follows almost the entire length of the same. Accompanying this suture, are found setae implanted in the dorsal region. Lateral lobe 286 (277 ± 7.3; n = 10) long and 24 (25 ± 2.9; n = 10) wide. Lateral lobe/gonocoxite ratio 1.17: 1 (1.20 ± 0.07; n = 10). Conical and pigmented aedeagus. Genital filament (Figure 4) 435 (425 ± 30.1; n = 10) long and 3.0 (3.0; n = 10) wide and genital pump 153 (150 ± 4.5; n = 10). Genital filament/genital pump ratio 2.84: 1 (2.83 ± 0.22; n = 10). Type of genital filaments slender and beak-shaped.

#### Allotype female

sand fly of medium size, measuring *ca*. 2,567 (2,574 ± 260.6; n = 10) in length. Coloration as in the males holotype and paratypes.

#### Head (Figure 5)

414 (422 ± 30.6; n = 10) long and 290 (301 ± 18.0; n = 10) wide. Head length/head width ratio 1.43: 1 (1.40 ± 0.10; n = 10). Clypeus 153 (168 ± 14.0; n = 10) long; clypeus length/head length ratio 0.37: 1 (0.40: 1 ± 0.01; n = 10). Eye 133 (142 ± 10.4; n = 10) long and 92 (85 ± 9.7; n = 10) wide; eye length/head length 0.32 (0.34: 1 ± 0.03; n = 10). Interocular distance 143 (146 ± 17.0; n = 10). Labrum-epipharynx (LE) 292 (306 ± 18.4; n = 10). LE/head length 0.71: 1 (0.73 ± 0.06; n = 10). Labial suture forming a fork. Antenna with simple and long ascoid and reaching the next flagellomere. Antennal formula AIII-AXV: 2 Antennomere lengths: AIII 235 (264 ± 18.8; n = 10); AIV 122 (128 ± 7.4; n = 10); AV 122 (128 ± 7.4; n = 10); AXV > AXVI (AXV > AXVI; n = 10). AIII, AIV, AXIV, AXV and AXVI with papilla; ratios: AIII/head length 0.57: 1 (0.63: 1 ± 0.05; n = 10); AIII/LE 0.80: 1 (0.86: 1 ± 0.06; n = 10). Palpal formula 1.4.3.2.5 (1.4.3.2.5; n = 10). Palpomere lengths: P1 51 (48 ± 4.6; n = 10); P2 153 (158 ± 8.1; n = 10); P3 173 (177 ± 10.8; n = 10); P4 112 (120 ± 8.4; n = 10); P5 442 (446 ± 20.5; n = 10). The Newstead spines implanted in the median region of the third palpomere. Cibarium with four posterior (horizontal) teeth developed and individualized, not fused in their base. The anterior (vertical) teeth are present in greater number in the regions lateral of the cibarium, starting from the two sides of the cibarium, and forming an arch with about four teeth larger vertical, situated below the posterior teeth (Figure 6). Sclerotized area is well defined and the sclerotized arch is complete. Unarmed pharynx. Lacinia of the maxilla with 7-9 external teeth in a single longitudinal row.

**Figure 5:**
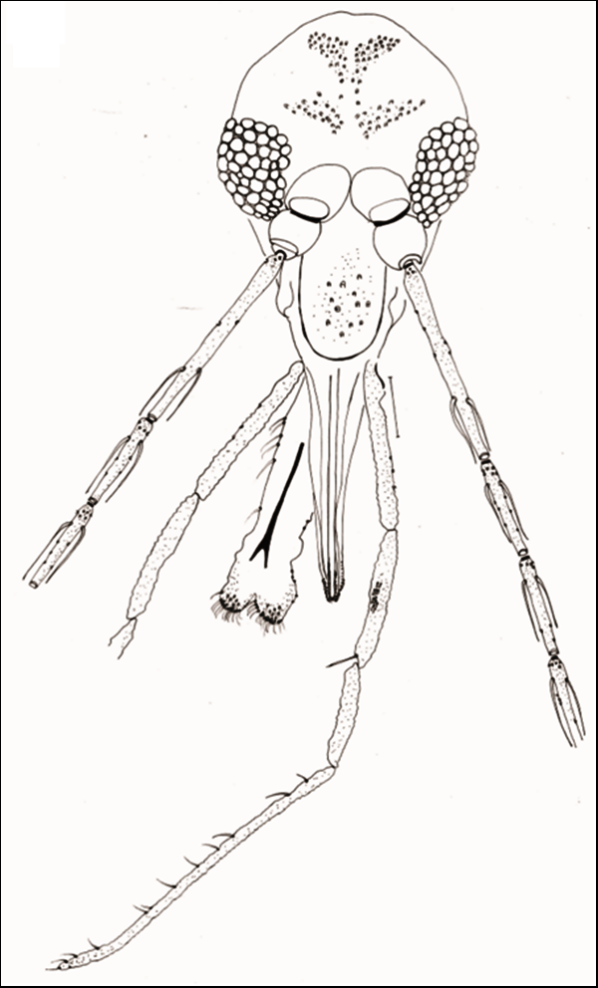
***Evandromyia spelunca *sp. nov. (Alotype female)**. Head, frontal view. Bar = 100 μm.

**Figure 6:**
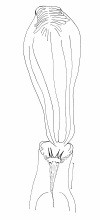
***Evandromyia spelunca *sp. nov. (paratype female)**. Pharynx and cibarium. Bar = 100 μm.

#### Cervix

Ventrocervical sensillae: present

#### Thorax

**Figure 7:**
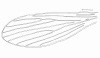
***Evandromyia spelunca *sp. nov. (paratype female)**. Wing. Bar = 250 μm.

Proepimeral setae present, 3-4 [(3-4; n = 2) (5-6; n = 3), (5-5; n = 2), (4-5; n = 1), (4-4; n = 1)] and anepisternal superior setae present, 10-11 [(15-15; n = 1), (20-20 n = 1), (13-14; n = 1); (14-15; n = 1), (17-18; n = 1), (11-13; n = 1), (16-17; n = 2)]; setae on the anterior region of the katepisternum present. Wing (Figure 7) measurement 2,029 (2,040 ± 141.9; n = 10) long and 442 (558 ± 69.1; n = 10) at maximum width. Length/width ratio 4.59:1 (3.66: 1 ± 0.37). Length of the vein sections: R_5 _1,311 (1,324 ± 103.4; n = 10); *alpha *414 (439 ± 69.9; n = 10); *beta *262 (266 ± 28.3; n = 10); *gamma *759 (755 ± 55.5; n = 10); *delta *110 (145 ± 38.2; n = 10). Legs, anterior, median and posterior, respectively: femur 773 (796 ± 59.0; n = 9), 718 (783 ± 70.6; n = 10) and 828 (886.6 ± 79.2; n = 8); tibia 842 (891 ± 74.4; n = 9), 994 (1,048 ± 80.8; n = 4) and 1,229 (1,273 ± 100.7; n = 8); tarsomere I 511 (570 ± 61.5; n = 9), 593 (625 ± 38.4; n = 10) and 690 (716 ± 33.3; n = 7); tarsomeres II+III+IV+V 662 (652 ± 56.7; n = 9), 649 (687 ± 56.2; n = 10) and 690 (735 ± 49.6; n = 7).

#### Abdomen

**Figure 8:**
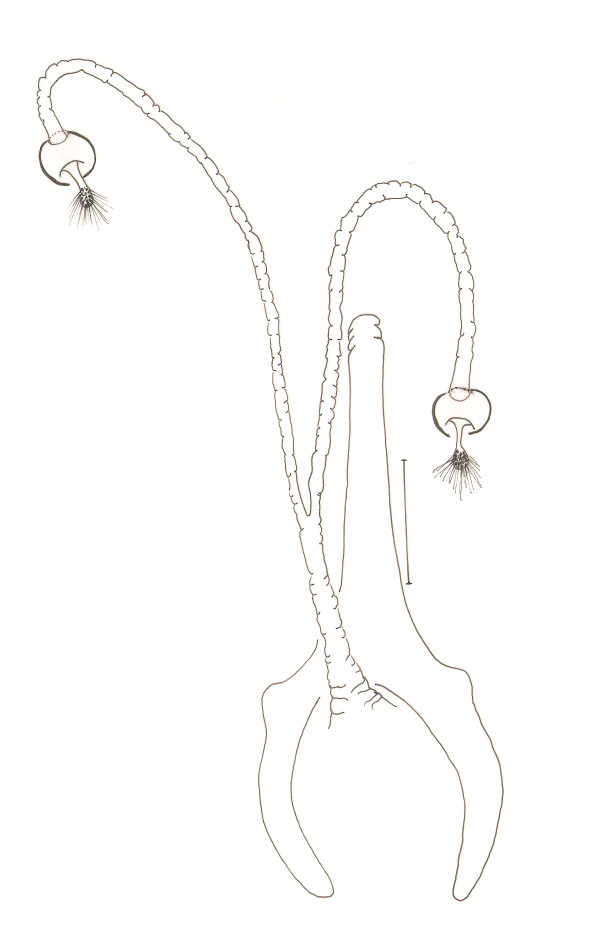
***Evandromyia spelunca *sp. nov. (paratypes female)**. Spermathecae. Bar = 100 μm.

spermathecae (Figure 8): 20 (20 ± 1.7; n = 10) long by 17 (17; n = 10) at maximum width. The body of the spermatheca is globular with diameters approximately equals to the others of group. The head of the spermathecae present some fine bristles inserted in the apex. The individual and common sperm ducts are smooth-walled, the latter being short compared to the first. The individual duct is 197 (199 ± 12.4; n = 8) in length and the common duct 47 (48 ± 2.7; n = 8). Cercus 119 (125 ± 15.7; n = 10) long.

#### Type-material

Hotype male (N. 89,458) and allotype female (N. 87,358) collected respectively on 30^th ^September 2009 and 26^th ^June 2008 with CDC light traps, model HP, in a limestone cave named "Gruta Rebenta Bombas", municipal district of Lassance, Minas Gerais state, Brazil (Carvalho, G.M.L. and colleagues), together with nine male paratypes (N. 87,715 collected on 28/08/2008; N. 89,459 to N.89,464 collected on 30 09 2009;N.89,465 collected on 26/05/2009 and 89,466 collected on 10/03/2010) and nine female paratypes (N. 86,800 collected on 17/04/2008; 86,970; and 87,011 collected on 21/05/2009; 87,158; 87,233;87,367; 87,368 collected on 26/06/2008; 87,403 and 87,405 collected on 26/05/2008). The type-material is deposited in the "Coleção de Flebotomíneos" of the "Instituto René Rachou/FIOCRUZ", Belo Horizonte, Brazil.

#### Etymology

The name *Evandromyia spelunca *sp. nov. has been given in regards to the meaning of cave in Latin.

## Results and Discussion

The morphological characters of the new species permit included in the *Evandromyia *genus, in the *cortelezzii *complex. This complex consists of three species: *Evandromyia corumbaensis *(Galati, Nunes, Oshiro & Rego, 1989), *Evandromyia cortelezzii *(Brethes, 1923) and *Evandromyia sallesi *(Galvão & Coutinho, 1940). These species are morphologically close and often confused, causing errors in its specific identification [5].

The males of the genus *Evandromyia *present the gonocoxite with a tuft of bristles, basal and compact; the paramere is simple or branched; the lateral lobe apex is thin and the females present the relationship between the lengths: clypeus/head wider than 1/3; eye/head less than 1/2; and spermathecae, with variable body. The *cortelezzii *complex belongs to the *cortelezzi *series of the *Barretomyia *subgenus. This series presents as main morphological characteristics: males, with the paramere and aedeagus simple; the gonostyle with upper external spine implanted in his middle, and lower spine, inserted in the basal third. Females with the globular spermathecae's body and the individual ducts are tubular [3]. *Evandromyia edwardsi *(Mangabeira, 1941) also belong to this series.

**Figure 9:**
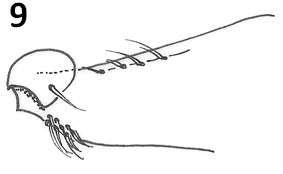
***Evandromyia sallesi *- Paramere**. Bar = 100 μm.

**Figure 10:**
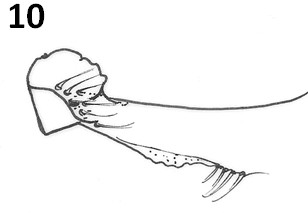
***Evandromyia cortelezzii *- Paramere**. Bar = 100 μm.

**Figure 11:**
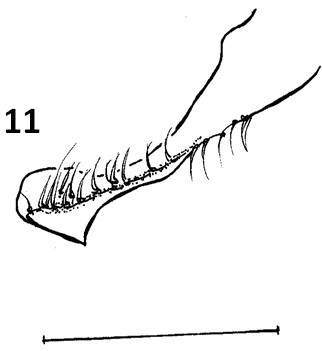
***Evandromyia corumbaensis *- Paramere**. Bar = 100 μm.

The new species can be separated from others of the *cortelezzii *complex through morphological characters. All the males of the complex are very close being separated by the following characteristics: paramere, which provides more similar aspect to *Ev. cortelezzii *and *Ev. sallesi*, however, with the internal suture, the curvature and the shape of the paramere, being enough to separate the new species from the other two closer (see Figures 3, 9 and 10). *Ev. sallesi *presents the paramere with the most consistent shape compared to all other species, so being the most robust (Figure 9). The suture that divides the inside of the paramere is more central and follows almost the entire length of paramere in the new species. This is more evident in the parameres of the *Ev. cortelezzii *and *Ev. corumbaensis*, the latter being the thinnest and convex paramere of all species of the complex (Figure 11). The lateral lobe is slightly longer than all other species of the complex. The base of the tuft presents a small pigmented area, being this characteristic common to the three other species of the *cortelezzii *complex. But with the tuft of the gonocoxite in the new species being slightly different from the others three species of the complex, which provides a more similar aspect to *Ev. corumbaensis*, both separated by the aspect of the bristles, more thinner in *Ev. corumbaensis*, and the tubercle that presents less setae in the new species.

**Figure 12:**
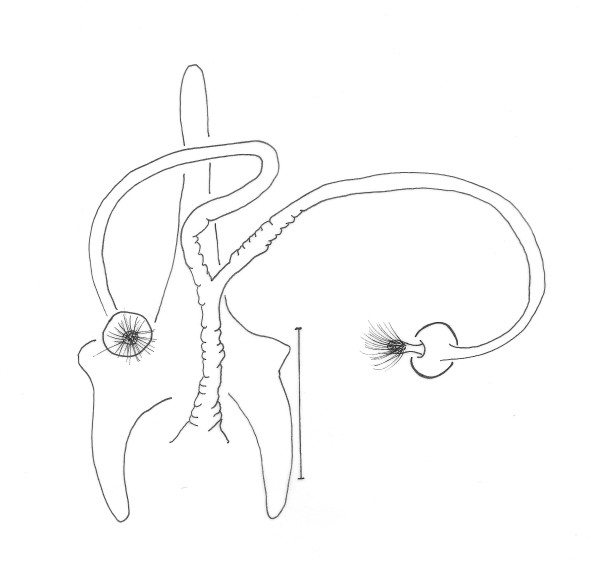
***Evandromyia sallesi *- Spermathecae**. Bar = 100 μm.

The separation of the females from the others of the complex is more confused, and was not always possible, usually when the spermathecae are not present or totally visible. However, the new species can be separated from the *Ev. cortelezzii *and *Ev. sallesi *(Figure 12) by spermathecae and characteristics of their ducts: individual and common, which are noticeably longer in the new species (Figure 8) and more similar to *Ev. corumbaensis*. Only characteristics of the cibarium were not enough to separate the females of the complex. We can utilize characteristics of the cibarium to separate the species of the complex, observing the distance between the vertical teeth implantation, and position of the arch that form the horizontal teeth, this distance being greater in the new species. Added to this, it is important to check the layout of vertical teeth in the new species, which appears in the form of an arc, starting at the sides of cibarium, from top to bottom, which usually causes the distance between the deployments mentioned above, higher in *Ev. spelunca *sp. nov.

Due to the difficulty of identifying the species of this complex, it became routine, with rare exceptions [6], to use geographic distribution for identify them in Brazilian regions. However, it was recently demonstrated that the three species of the complex occur in all states of the Center-west Region, and only *Ev. corumbaensis *is restricted to that region. The species of this complex that present the greater distribution in the country is *Ev. cortelezzii*, demonstrating its omission from the lists of species in several states due *Ev. sallesi*, which has more restricted distribution than the one quoted in the literature [7]. Thus, we do not suggest the use of geographic distribution to identify the species of this complex, but we can suggest the observation of its occurrence in caves, beyond the morphological characteristics, since the new species had their captures practically restricted to inside the cave studied, showing preference for this environment.

It is still important to emphasize that the new species is sympatric with *Ev. sallesi *inside or outside the cave, however the latter one are always captured inside the cave in less number than outside the cave (data not shown).

## Conclusions

The present study adds a new species, namely Ev. spelunca sp. nov., to the Brazilian phlebotomine sand fly fauna, presenting characters for its separation from members of the cortelezzii complex, to which it belongs With the description of the new species, now the *cortelezzii *complex contains 4 species, belonging to the *cortelezzii *series of the *Barretomyia *subgenus, *Evandromyia *genus.

## Competing interests

The authors declare that they have no competing interests.

## Authors' contributions

GMLC, RPB, CCS and JDAF participated in morphological analysis and taxonomic discussion of the specimens. GMLC and CCS did drawing and measurements of the new species. GMLC, JDAF and RPB drafted the manuscript. All authors read and approved the final version of the manuscript.
